# Exosomes as Regulators of Macrophages in Cardiovascular Diseases

**DOI:** 10.3390/biomedicines12122683

**Published:** 2024-11-25

**Authors:** Marina Soriano-Cruz, Wendy Guadalupe Vázquez-González, Paula Molina-Vargas, Alejandro Faustino-Trejo, Adriana Karina Chávez-Rueda, María Victoria Legorreta-Haquet, Sergio Roberto Aguilar-Ruíz, Luis Chávez-Sánchez

**Affiliations:** 1 Unidad de Investigación Médica en Inmunología, Hospital de Pediatría, Centro Médico Nacional Siglo XXI, Instituto Mexicano del Seguro Social, Mexico City 06720, Mexico; marinasscc@gmail.com (M.S.-C.); wendisya@live.com.mx (W.G.V.-G.);; 2 Unidad de Investigación Médica en Enfermedades Metabólicas, Hospital de Cardiología, Centro Médico Nacional Siglo XXI, Instituto Mexicano del Seguro Social, Mexico City 06720, Mexico; 3 Facultad de Medicina y Cirugía, Universidad Autónoma Benito Juárez de Oaxaca, Oaxaca 68020, Mexico

**Keywords:** atherosclerosis, myocardial infarction, macrophage inflammation, exosome

## Abstract

Macrophages in atherosclerosis and myocardial infarction have diverse functions, such as foam cell formation and the induction of an inflammatory response that promotes ventricular dysfunction in the heart. Exosomes are small vesicles released by many different types of cells, such as macrophages, dendritic cells, platelets and other immunoregulatory cells, that facilitate communication with other cells, modulating the biological functions of recipient cells. Exosomes offer a novel therapeutic approach for the polarization of macrophages involved in cardiovascular diseases. In this review, we provide an overview of the biological role of macrophages in atherosclerosis and myocardial infarction and the effects of exosomes on these cells as therapeutic agents in the disease.

## 1. Introduction

Cardiovascular diseases (CVDs) remain the leading cause of death worldwide, accounting for 18 million deaths in 2023 according to the World Health Organization. CVDs are a group of disorders of the heart and blood vessels [1]. Most CVDs originate from atherosclerotic disease, which is a chronic inflammatory condition involving multiple arteries throughout the body. Therefore, atherosclerosis is the most common underlying cause of carotid artery disease, coronary artery disease, and arterial disease [2].

Atherosclerotic plaques tend to form in the internal curvatures and branching points of the arteries, where laminar flow is disturbed or insufficient to maintain the normal state of the endothelium, increasing vascular permeability. Under these conditions, low-density lipoprotein (LDL) accumulates in the intima of the artery and is susceptible to modifications such as oxidation, generating oxidized LDL (oxLDL) and starting the development of atherosclerotic plaque [3]. Several cell types contribute to atherosclerotic plaque formation, including macrophages, particularly inflammatory macrophages that accumulate in atherosclerotic plaques and influence inflammatory activity, and during infarction inflammatory macrophages initiate the inflammatory response through cytokines such as IL-1β, free radicals, phagocytosis, and efferocytosis, which allow healing of the infarcted tissue. However, macrophages can contribute to adverse remodeling in infarction via the deposition of extracellular matrix proteins, impacting cardiac function, which manifests as diastolic and/or systolic dysfunction, the development of arrhythmias, and myocardial ischemia [4].

Finding new ways to improve the prognosis of myocardial infarction is important. In this context, exosomes are extracellular lipid bilayer vesicles that are secreted by most eukaryotic cells. Exosomes are involved in the regulation of numerous biological processes, including tissue repair, immune modulation, and disease progression, which gives them great potential as bioindicators and therapeutic agents. These functions of exosomes originate from their content, i.e., a wide variety of bioactive molecules, such as proteins, lipids, nucleic acids, and cytokines, which mediate intercellular communication [5,6]. The functional activity of exosomes derived from different types of cells in CVDs is currently under investigation [7]. However, understanding the role of cell-derived exosomes in the pathophysiology of atherosclerosis and infarction, as well as their therapeutic potential in these conditions, is essential. In this review, we examine the biological effects of exosomes from several cell sources, including those derived from macrophages and other target cells involved in CVDs.

## 2. Atherosclerosis and Myocardial Infarction

CVDs are the leading cause of death worldwide and are a group of heart and blood vessel disorders. Atherosclerosis is recognized as a chronic inflammatory disease and is the starting point of many diseases affecting the carotid and coronary arteries. This disease begins with endothelial dysfunction caused by various stimuli, such as the interaction of endothelial cells (ECs) with oxLDL, proinflammatory cytokines, and turbulent flow. In this state, endothelial cells become unstable; overexpress adhesion molecules such as LOX-1, PECAM-1, ICAM-1, and VCAM-1; and release proinflammatory cytokines such as mCSF and CCL-2, attracting, retaining, and allowing the transmigration of various immune cells, such as monocytes [3,4].

**Figure 1:**
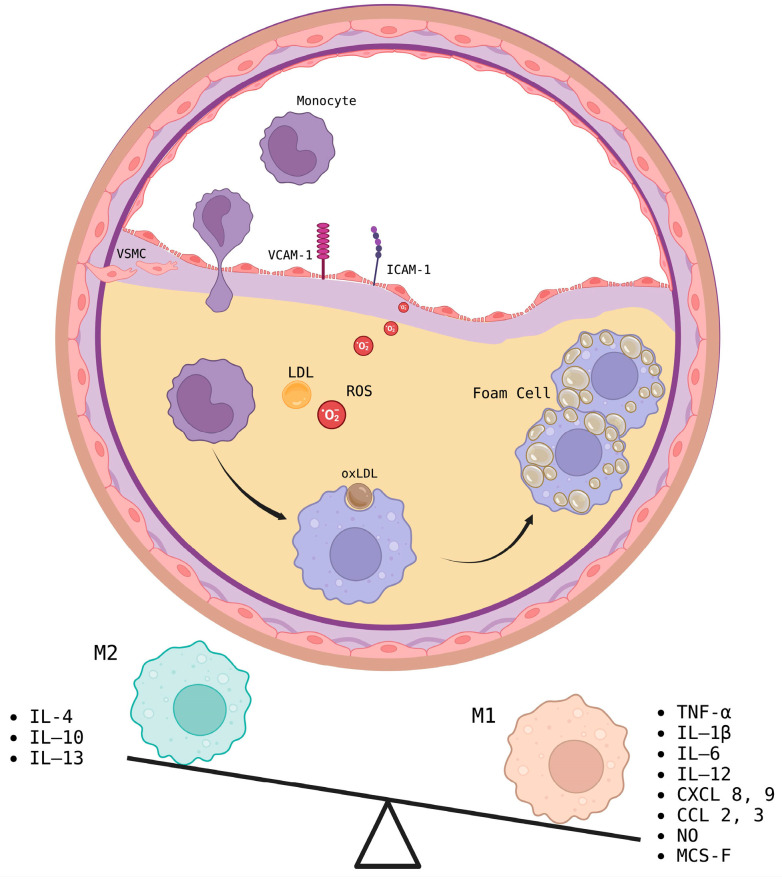
Macrophages in atherosclerosis. Monocytes are recruited to the atherosclerotic plaque by CCL2 as well as ICAM and VCAM-1 and are internalized into the intima, where LDL is modified by oxygen free radicals. Monocytes differentiate into macrophages that uptake oxLDL, transforming into foam cells that accumulate in the plaque. The inflammatory microenvironment causes M2 macrophages (inflammation resolving) that predominate in early plaque to be replaced by M1 macrophages (inflammatory) as the plaque progresses.

In the intima of the endothelium, monocytes differentiate into macrophages, and LDL particles are retained by proteoglycans, leading to their oxidation. These oxLDLs are then phagocytosed by macrophages, leading to foam cell formation and the secretion of proinflammatory cytokines such as IL-1, TNF-α, and reactive oxygen species (ROS), causing an imbalance of vasoconstrictors and vasodilators as the availability of NO is limited (Figure 1). Foam cells accumulate, forming a fatty streak [3,4]. During fatty streak formation, foam cells may undergo necrosis or apoptosis, and the accumulation of cell contents results in the formation of a necrotic core. This process also leads to the release growth factors such as platelet-derived growth factor (PDGF-1), basic fibroblast growth factor (bFGF), and transforming growth factor-beta (TGF-β), promoting the proliferation and migration of smooth muscle cells, as well as collagen and elastin deposition, forming a fibrous cap that protects the necrotic core; this atheroma alters blood flow, causing ischemia [3,4].

The pro-atherogenic environment stimulates the growth of the intima and endothelium, creating a hypoxic environment within the plaque, activating HIF-1, and sending a survival signal to endothelial cells, which secrete vascular endothelial growth factor (VEGF), stimulating angiogenesis of blood vessels in the outer layer of major arteries and promoting the formation of additional vessels within the vascular wall. At this stage, the atherosclerotic plaque grows larger and is known as a fibroatheroma [3,4]. The plaque can be stable or unstable; it is considered unstable when macrophages secrete matrix metalloproteinases that degrade the fibrous cap [8].

MI begins when an unstable plaque ruptures, exposing the necrotic core content to the bloodstream and forming a thrombus that partially or completely obstructs blood flow, causing hypoxia. The immune response during infarction consists of three phases: inflammation, proliferation, and maturation. The response begins with the recognition of damage-associated molecular patterns (DAMPs) due to cell death, and cell contents are identified by macrophages in the affected area; these macrophages release chemokines and proinflammatory cytokines, recruit neutrophils and monocytes, and mount a proinflammatory signal that peaks at three days. The inflammation phase is followed by a proliferation phase with the deposition of granulation tissue and, finally, the maturation phase, involving extracellular matrix remodeling, resulting in significant changes in the size, shape, and function of the ventricles in the months following MI. Macrophages are highly involved in all three phases, presenting a proinflammatory phenotype to mount repair signals. Two weeks post-MI, macrophage populations decrease, and they are recruited to the remote myocardium to stimulate remodeling [9].

## 3. Macrophages

Macrophages participate in inflammation, angiogenesis, wound healing, immune regulation, and homeostasis [4]. Macrophages can be classified into M1-like macrophages with a proinflammatory profile, also known as classically activated or proinflammatory, and M2-like macrophages with a reparative profile, also known as alternatively activated or anti-inflammatory [10]. Macrophages are present in all human organs, and their functions depend on the organ and environment [4]. In the heart, macrophages can aid in angiogenesis, maintaining heart rhythm, and developing various pathologies, but their role depends on their polarization (M1 macrophages or M2 macrophages), which is influenced by the surrounding environment [11].

M1-like macrophage polarization is induced by pathogen-associated molecular patterns (PAMPs) molecules such as lipopolysaccharide (LPS), interferon-γ (IFN-γ), and granulocyte-macrophage colony-stimulating factor (GM-CSF), which trigger the PI3K–AKT–mTOR–HIFα pathway. M1-like macrophages in atherosclerosis release proinflammatory cytokines, such as tumor necrosis factor-alpha (TNF-α), IL-1β, IL-6, nitric oxide (NO), IL-12, IL-23, CXCL8, CXCL9, CXCL10, CXCL11, CXCL16, CCL2, CCL3, and CCL5 [12,13]. The markers used to identify this macrophage subtype include CD86, CD38, iNOS, TNF-α, MCP-1, and pAKT. These genes positively regulate glycolysis, the pentose phosphate pathway, and fatty acid synthesis [14].

M2-like macrophage polarization is induced by IL-4 and IL-13 via the JAK-STAT, PPAR, AMPK, and/or TGF-β pathways and by glucocorticoid receptor overexpression. The markers expressed include CD206, Arg-1, Relm-α, Chi313, and PPAR-γ. This cell type positively regulates the oxidative phosphorylation and β-oxidation pathways. M2-like macrophages release IL-10 and do not secrete proinflammatory cytokines [14].

## 4. Macrophages in Atherosclerosis and Myocardial Infarction

In early atherosclerotic lesions, a high density of M2-like macrophages is present, and M1-like macrophages predominate in advanced lesions. M1-like macrophages are distributed in the rupture-prone shoulder regions of plaques. In contrast, most M2-like macrophages are located in vascular adventitial tissue as well as in stable plaques. In atherosclerosis, M1-like macrophages cause sustained inflammation, infiltrating the plaque base in early stages, whereas in advanced stages, they are located around the necrotic core, promoting the polarization of more M2-like macrophages to M1-like macrophages and affecting M2-like macrophages via cytotoxic effects [15]. M1-like macrophages recognize oxLDL through TLR4 and CD36, increasing the levels of proinflammatory cytokines such as IL-1β, IL-8 and IL-6; the expression of HLA-DR and CD86; and the proliferation of T cells [16]. This inflammatory microenvironment induced by IL-1β induces endothelium activation and the recruitment of monocytes and the activation of M1-like macrophages [17]. The interaction of macrophages with oxLDL through TLR4 and CD36 allows the formation of foam cells, which are key in the development of atherosclerosis. Additionally, oxLDL exerts a cytotoxic effect on M2-like macrophages, unlike M1 macrophages, which are more resistant to oxLDL lipotoxicity, suggesting that oxLDL could contribute to M1-like macrophage activation and M2-like macrophage killing, thus favoring other macrophage subpopulations [18]. Additionally, C-reactive protein (CRP) induces M1-like macrophage polarization, accompanied by increased levels of TNF-α and IL-1β. Surprisingly, in the presence of CRP, M2-like macrophages secrete proinflammatory cytokines, indicating the conversion of M2-like macrophages to M1-like producers of TNF, IL-12, and IL-23 [14]. This chronic inflammatory microenvironment allows the progression of a stable plaque into an unstable plaque [19]. This event is caused by cholesterol accumulation in the intima, which preserves polarization signaling to M1-like macrophages, increasing inflammation [20,21,22]. In advanced stages of atherosclerosis, M1-like macrophages are present in the necrotic core and secrete high levels of IL-12, IL-23, reactive oxygen species (ROS), and matrix metalloproteinases (MMPs) (MMP1, MMP2, MMP9, and MMP10) [12,13]. The rupture of the fibrous plaque allows the contents of the necrotic core to come into contact with the blood stream, inducing the formation of a thrombus that partially or totally obstructs blood flow, resulting in MI.

In the early stage of MI, chemokines such as CCL2 are released, attracting Ly-6C^high^ monocytes, which are predominant in early MI, as well as tissue-resident CCR2+ macrophages, which are critical drivers of monocyte mobilization and recruitment to the heart, where they differentiate into M1-like macrophages. CCR2+ macrophages express increased levels of genes related to inflammatory chemokines, such as *Cxcl2*, and cytokines, such as *Il1β*, as well as genes associated with TNF and NF-κB [23]. During MI, macrophages respond to DAMPs released by cardiomyocytes as well as to IFN-γ-derived signals, resulting in the activated M1-like macrophages that secrete IL-1β, IL-6, TNF-α, and reactive oxygen species (ROS), increasing their profibrotic activity and phagocytic profile [12,13]. If these inflammatory conditions are maintained by macrophages in the myocardium, deterioration of LV systolic function and altered LV chamber dimensions can occur, further aggravating myocardial cell injury. Macrophages actively remove dead cells that express phosphatidylserine and are recognized by TAM receptors such as myeloid-epithelial-reproductive receptor tyrosine kinase (MerTK) [9,24,25] during the initial process of cardiac repair. M2-like macrophages play crucial roles in tissue repair postinfarction; they secrete anti-inflammatory cytokines such as IL-10 and the growth factors TGF-β and VEGF, promoting tissue healing and remodeling by stimulating angiogenesis. M2-like macrophages in mice are derived from Ly-6C^low^ monocytes and promote tissue healing by activating fibroblasts and promoting extracellular matrix deposition. At six weeks postinfarction, macrophages concentrate in the remote myocardium to stimulate remodeling. Macrophages are implicated in postinfarction cardiac fibrosis by stimulating fibroblast differentiation into myofibroblasts via TGF-β signaling, causing endothelial-mesenchymal transition (EndoMT) in endothelial cells, perpetuating signaling, and leading to heart failure [4,9].

Macrophages are innate cells that are highly active in all stages of atherosclerosis and after MI. The differentiation and activation of macrophages are crucial in these pathophysiological conditions. Therefore, studying macrophage polarization is crucial for identifying promising therapeutic targets. Currently, small extracellular vesicles called exosomes, which are secreted by various cells, can directly influence macrophage polarization, turning macrophages into therapeutic targets.

## 5. Origin and Function of Exosomes

Exosomes are a type of extracellular vesicle secreted by all cell types in the human body (endothelial cells, mesenchymal stem cells, T cells, B cells, macrophages, dendritic cells, and natural killer cells) and have a diameter of 30–150 nm [5]. Exosomes have been found in plasma, urine, semen, saliva, breast milk, tears, bile, gastric acid, lymph, synovial fluid, amniotic fluid, serum, cerebrospinal fluid, and bronchial fluid [26].

Exosomes originate from the initial endosomes formed by membrane invagination, followed by the accumulation of bioactive substances in early sorting endosomes. Endosomes become late endosomes when they interact with the endocytic sorting complex and transport-associated proteins. Late endosomes mature into multivesicular bodies, which fuse with the cell membrane to release their contents outside the cell. The phospholipid bilayer membrane of exosomes contains lipid structures such as ceramide and cholesterol, along with the endosomal sorting complex required for transport (ESCRT), which are fundamental for exosome release and generation [5]. There are also ESCRT-independent pathways, such as the tetraspanin pathway, the Rab GTPase superfamily pathway via the Rab protein, and the ceramide sphingolipid synthesis pathway, which can be synergistic with ESCRT [27].

**Figure 2:**
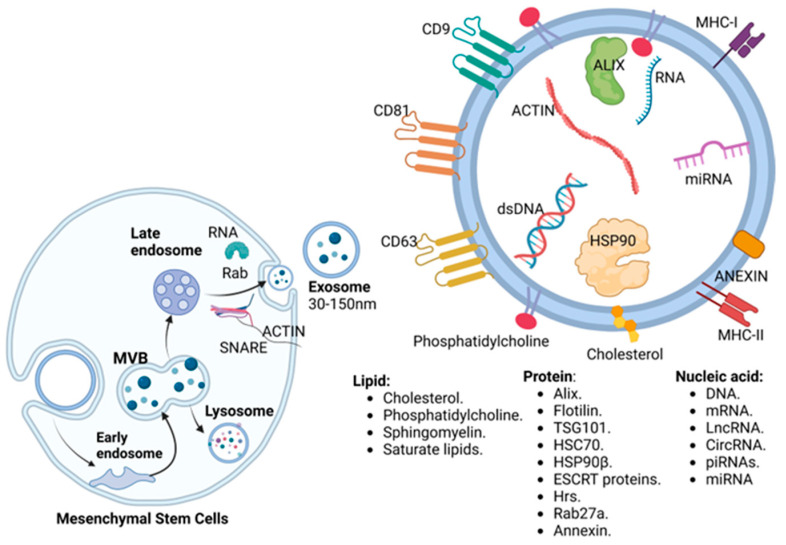
The formation of exosomes begins with the invagination of the membrane, forming the early endosomes. Later, they acquire cell properties, thus reaching maturation; in this phase, endosomes are called multivesicular bodies. Finally, exosomes can be degraded by lysosomes, or released through pathways independent of or dependent on ESCRT, Rab GTPases, and SNARE complexes, to deliver their content to other neighboring or distant cells. Exosomes usually measure from 30 to 150 nm, and their content usually contains lipids that provide stability and resistance for transport, a wide variety of proteins, and nucleic acids, with miRNAs being the most relevant for communication with other cells.

Exosomes transport lipids, proteins, metabolites, dsDNA, and RNA, and their function depends on the exosome content and the cell of origin. Generally, exosomes contain cholesterol, phosphatidylcholine, sphingomyelin, and saturated lipids, providing rigidity and resistance to degradation and protecting protein and nucleic acid contents [28,29]. Protein transport is characteristic of each type of extracellular vesicle (EV). Exosomes are highly enriched with proteins with multiple functions, such as CD9, CD63, and CD81, which are membrane proteins specific to exosomes; accessory proteins such as ALIX, flotillin, TSG101, HSC70, HSP90β, and ESCRT proteins; associated proteins such as Hrs, Rab27a, and annexin [30,31]; MHC class I molecules; and MHC class II molecules. EVs heterogeneously transport nucleic acids; however, there are distinctive features. Exosomes contain a relatively high load of mRNAs, approximately 500 miRNA molecules per exosome, double-stranded DNA (dsDNA), and various noncoding RNAs such as circular RNAs (circRNAs), PIWI-interacting RNAs (piRNAs), and long noncoding RNAs (lncRNAs) [32,33]. Metabolite contents include cyclic alcohols, amino acids, vitamins, nucleosides, carnitine, organic acids, aromatic compounds, and sugars with their conjugates [34] (Figure 2).

Exosomes have been shown to facilitate cell communication, tumor maintenance and progression, and immune response activation by either activating receptors on neighboring cells or fusing with cells to release their contents; this can occur with nearby cells or distant cells [35,36]. Exosomes can also serve as biomarkers for certain diseases [36]. The functions of exosomes depend on their content, which is provided by the effector cell and the microenvironment. For example, exosomes derived from dendritic cells (DCs) induce specific immune responses due to MHC class I and II expression, and exosomes with high ceramide phosphate contents promote inflammation [37,38].

The function of exosomes depends not only on their content or the microenvironment but also on the mechanisms and pathways associated with the uptake of exosomes by recipient cells. These processes can include clathrin-mediated and caveolae-dependent endocytosis, macropinocytosis, lipid raft participation, membrane fusion at the cell surface, and specific exosome uptake by the cell. Once the interaction between the exosome and the recipient cell occurs, the content of the exosome is released, triggering a signaling cascade. In this context, clathrin-mediated endocytosis involves the interaction of exosome ligand-receptor complexes coated with clathrin. The clathrin coat deforms the membrane, forming larger vesicles that enclose the exosome for internalization. After internalization, the clathrin coat is shed, and the vesicle fuses with the endosome, releasing its content. This pathway has been shown to facilitate efficient genetic information (miRNA) and protein delivery, activating signaling pathways [39,40,41].

Exosomes are nanoscale membrane vesicles with the exceptional ability to target specific cells or tissues; they may mediate the horizontal transfer of genetic material via interactions with surface adhesion proteins, resulting in modified biological activities of recipient cells [42]. Exosomes have a wide spectrum of functions in various cell types and diseases. Studies in mice have shown that some exosomes can directly deliver mRNA to recipient cells, especially under the stimulation of acute or chronic infection [43]. These exosomes may indirectly activate T cells by interacting with antigen-presenting cells and may also promote the proliferation of CD4^+^ T cells [44]. Exosomes released by stem cells have the capacity to inhibit inflammasome activation through the release of their contents, mainly miRNAs, which prevent the dissociation of NF-kB from IkB, thus inhibiting the nuclear translocation of NF-kB and reducing the expression of target genes such as *NLRP3*, *pro-IL-1β*, *pro-IL-18*, and *TNF-α.* Additionally, they reduce the conversion of procaspase-1 to caspase-1, inhibiting the secretion of the cytokines IL-1β, IL-18 and TNF-α by preventing the formation of the inflammasome complex via PRR, ASC, and procaspase-1. All this signaling is activated and enhanced upon the receipt of bioactive molecules from exosomes derived from immune cells [45]. For example, circular RNA (circRNA) derived from the *ATP8A1* gene (circTP8A1) induces macrophage differentiation to M2 via the circATP8A1/miR-1-3p/STAT6 axis, favoring tumor progression in gastric cancer [46].

## 6. Effects of Exosomes on the Regulation of Macrophages

**Figure 3:**
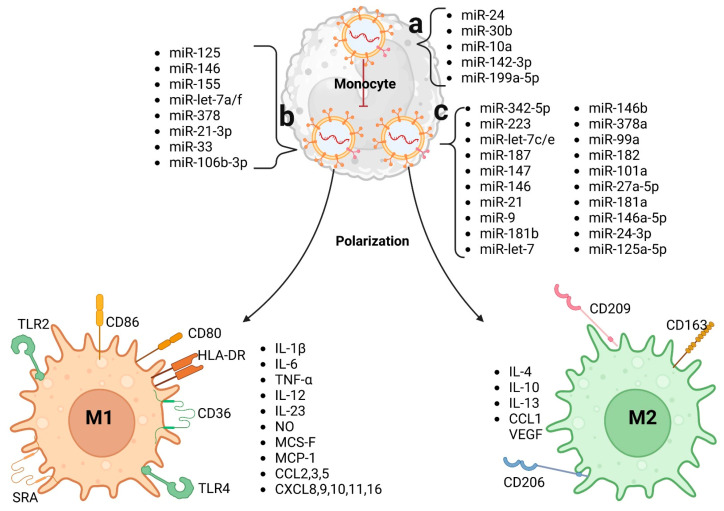
Effect of exosomes on monocytes and macrophages. (**a**) The influence of exosomes on macrophages starts from monocytes. Exosomes can provide monocytes with miRNAs that inhibit macrophage differentiation. Exosomes can provide miRNAs that stimulate macrophage polarization to (**b**) M1 macrophages or (**c**) M2 macrophages.

Exosomal miRNAs are essential for the communication of exosomes with macrophages, influencing processes intrinsic to these cells in several diseases, such as CVDs. In 2018, it was revealed that miRNAs such as miR-24, miR-30b, miR-10a, miR-142-3p, and miR-199a-5p inhibit the differentiation of monocytes into macrophages, whereas miR-125, miR-146, miR-155, miR-let-7a/f, and miR-378 induce the differentiation of macrophages to the M1-like phenotype. For M2-like macrophage polarization, miRNAs such as miR-342-5p, miR-223, miR-let-7c/e, miR-187, miR-147, miR-146, miR-21, and miR-9 are required [47]. More miRNAs have been discovered, and the mechanisms by which they influence diseases are being studied more thoroughly (Figure 3). In addition to miRNAs, exosomes contain proteins. In this sense, exosomes can contain CSF-1, CCL2, FTH, FTL, and TGF-beta, which promote the polarization of macrophages to the M2-like macrophage phenotype [48]. It has also been shown that EPC-derived exosomes contain antioxidant properties as well as elevated levels of IL-10 [49]. Another exosomal component that may influence the proinflammatory profile of macrophages is sphingolipids, due to TLR4 signaling [50].

## 7. Exosome–Macrophage Communication in Atherosclerosis

In the development and progression of atherosclerosis, the polarization of macrophages is vital, as are the factors that influence these cells. Exosomes can influence the differentiation of macrophages to either a proinflammatory or anti-inflammatory profile through the internalization of their contents into these cells. In rats with type 2 diabetes mellitus, exosomes derived from M2-like macrophages have been shown to stimulate the conversion of macrophages from the M1-like macrophage to M2-like macrophage phenotype through the PI3K/AKT pathway [51]. In the proinflammatory environment of obesity, macrophages release exosomes containing miR-155 that directly affect endothelial progenitor cells [52]. In an atherogenic environment, exosomes derived from macrophages present in plaques promote plaque formation due to the reduction in naive macrophages caused by miR-146a, which negatively regulates *IGF2BP1*, diminishing growth and tubular structure formation in endothelial cells [53,54]. Exosomal miR-21-3p from nicotine-stimulated macrophages promotes atherosclerosis progression by inhibiting the *PTEN* pathway, which stimulates the migration and proliferation of vascular smooth muscle cells [55]. In an ApoE^−/−^ mouse model, M2-like macrophages that internalized miR-let-7 attenuated atherosclerosis [56], whereas the opposite occurred when miR-33 was internalized by macrophages; miR-33 increased M1-like macrophage gene expression and upregulated endothelin-1 (ET-1) expression, promoting atherosclerosis in ApoE^−/−^ mice [57].

Macrophages not only function as receptors in communication with exosomes but also release exosomes to communicate with surrounding cells. The decrease in exosomal *GAS5* derived from the THP-1 monocytic cell line regulates the apoptosis of macrophages and vascular endothelial cells in atherosclerosis [58]. During foam cell formation, THP-1 monocytes release exosomes containing high levels of miR-4532, which are internalized by endothelial cells. This miRNA inhibits specificity protein 1 (SP1), activating NF-kB P65 signaling and promoting the release of ICAM-1 and VCAM-1, thus causing the progression of atherosclerosis [59]. Exosomal miRNAs, including miR-146b, miR-378a, and miR-99a, promote differentiation toward M2-like macrophages; reduce inflammation and hematopoiesis through the NF-kB/TNF-α pathway; and have stabilizing effects on atheromas and reduce necrotic lesions [60]. In vitro assays have shown that exosomal LncNEAT1 derived from endothelial cells promotes bone healing by alternatively activating macrophages through the *DDX3X*/NLRP3 pathway [61]. miR-10a from extracellular vesicles derived from endothelial cells inhibits the differentiation of monocytes into macrophages [62]. Table 1 lists miRNAs associated with atherosclerosis.

## 8. Exosome–Macrophage Communication in Ischemia–Reperfusion

In a mouse model of ischemia-reperfusion, exosomes derived from mesenchymal stem cells containing miRNA-182 provided a cardioprotective effect by inhibiting the transcription of TLR4, leading to the increased proliferation of M2-like macrophages [63]. Additionally, miRNA-181a from exosomes derived from mesenchymal stem cells attenuates the inflammatory response and improves therapeutic capability [70]. Exosomal miR-27a-5p, miR-182, and miR-101a derived from cardiospheres reduce inflammation, the area affected by injury, and macrophage infiltration; improve cardiac function; and increase M2-like macrophage polarization [64]. Plasma exosomes from infarcted patients have low miR-26b-5p expression, thus inhibiting ferroptosis through the *SLC7A11*/GSH/*GPX4* pathway, enhancing cardiac function and reducing the damaged area [49].

In a balloon injury model, exosomes from endothelial progenitor cells promoted the proliferation, migration, and formation of vascular endothelial cells while negatively regulating apoptosis through the *Bcl2/Bax*/Caspase-3 pathway [65]. In ischemic disease, exosomal miR-21 from adipose-derived stem cells has been shown to activate macrophages in an alternative manner, exhibiting proangiogenic effects and mediating *CSF-1R* activation through the PI3K/Akt pathway [66]. Hypoxia–reoxygenation mediates macrophage polarization towards the M1-like macrophages, which mediate cardiomyocyte pyroptosis through exosomal miR-29a transfer by targeting myeloid cell leukemia-1 [71]. Table 1 lists miRNAs associated with ischemia–reperfusion.

## 9. Exosome–Macrophage Communication in Myocardial Infarction

**Figure 4:**
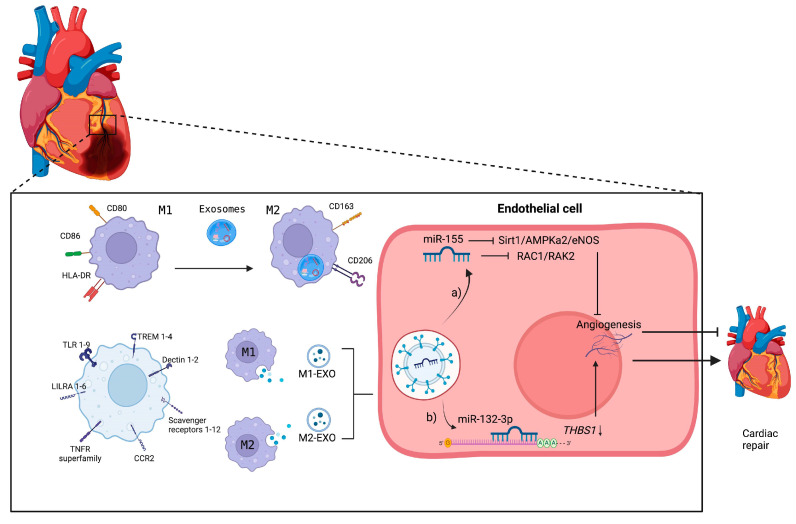
Exosomes and their influence on macrophages in the postinfarction period. After a heart attack, a microenvironment rich in exosomes released from cardiomyocytes, fibroblasts, endothelial cells, among others, prevails; these exosomes contain essential information to stimulate or stop cardiac repair. Macrophages can capture the exosomes that release their content in macrophages, stimulating the polarization of macrophages towards M1 and M2 or, failing that, from M1 to M2. Both types of macrophages release exosomes that stimulate or regulate cardiac repair: (**a**) M1-like macrophage-derived exosomes may contain elevated miR-155, which inhibits angiogenesis through the *Sirt1/AMPKa2/eNOS* and RAC1/RAK2 pathways, preventing cardiac repair, while (**b**) M2 exosomes can provide miR-132-3p, which binds to the 3’UTR of *THBS1* mRNA, preventing the overproduction of this protein, allowing cardiac repair signaling through angiogenic stimulation.

In patients with MI, exosomes derived from cardiomyocytes contain high concentrations of miR-146a-5p, indicating both proinflammatory and anti-inflammatory activity; this is due to the promotion of M1-like macrophage polarization and angiogenesis in macrophages [67]. This effect has been corroborated in an in vitro model of exosomes derived from cardiomyocytes with acute myocardial infarction cocultured with RAW264.7 cells, which promoted macrophage polarization toward the M2-like macrophage phenotype, similar to the findings for such cells cultured under hypoxic conditions [68]. During myocardial infarction, cardiomyocytes undergo ferroptosis. Exosomes from these cells influence the differentiation of macrophages to the M1-like macrophage phenotype by activating the Wnt/β-catenin signaling pathway via the expression of miR-106b-3p [69]. After infarction, M1-like macrophage cells release a variety of exosomes with an antiangiogenic profile, inducing cardiac dysfunction through miRNAs, with miR-155 being the most highly expressed and internalized by endothelial cells [59]. Similarly, M2-like macrophage exosomes deliver miR-132-3p to endothelial cells and enhance the angiogenic ability of endothelial cells by downregulating *THBS1* expression, thereby promoting angiogenesis after MI [72] (Figure 4). In addition, some exosomes during MI negatively affect cardiac regeneration. Exosomes derived from M1-like macrophages highly express miRNA-155, which, when transferred to cardiac fibroblasts, inhibits cardiac fibroblast proliferation by downregulating the expression of son of sevenless 1 and promotes inflammation by decreasing the expression of the suppressor of cytokine signaling 1 [73]. Table 1 lists the miRNAs associated with MI.

## 10. Macrophages as Therapeutic Targets

Macrophages have many functions in the cardiovascular system, such as regulating inflammation and fibrosis. Regulating the function of macrophages is a feasible strategy for the treatment of CVDs [74]. Given that MSCs might regulate the inflammatory immune response, the transplantation of bone marrow MSCs (BM-MSCs) into the infarcted area of the heart is an interesting approach for regenerating the myocardium [75]. Bone marrow mesenchymal stem cells increase the expression of markers of the M2-like macrophage phenotype (CD14, CD163, and CD206) and cytokines such as IL-10 and IL-1Ra in M1-like macrophages, favoring the induction of regulatory T cells [76]. Exosomes derived from BMS-MSCs transport high levels of miR-301, inhibiting cardiac autophagy by promoting the overexpression of P62, which inhibits the expression of LC3-II/LC3-I [77]. Exosomes derived from umbilical cord mesenchymal stem cells contain miR-24-3p, which promotes damage mitigation by M2-like macrophages during MI [78]. Exosomes derived from the C2C12 cell line of myoblasts under hypoxic conditions contain high concentrations of miR-29a, demonstrating a regulatory profile in tissue fibrosis after myocardial infarction in rats [79].

Exosomes derived from mesenchymal stem cells have positive effects on the repair of endothelial damage. Under this premise, Hu et al. (2021) [80] designed an ROS-sensitive MSC-derived exosome-releasing stent that has positive effects on the proliferation and migration of endothelial cells, as well as on inflammation and angiogenesis. MSC-derived exosomes have also been used as postinfarction treatments. Gong et al. (2024) [81] evaluated the potential of MSC-derived exosomes pretreated with nicorandil for cardiac repair and reported positive effects, as this treatment promoted the differentiation of macrophages to an anti-inflammatory profile through miR-125a-5p, which activated the *TRAF6/IRF5* pathway [82].

Platelet exosomes are secreted in large amounts during platelet activation and can regulate thrombosis through multiple pathways, involving platelets, endothelial cells, and inflammation [83]. Platelet exosomes induced a loss of surface CD36 on macrophages, reduced cholesterol loading, and modified LDL in macrophages, consequently leading to the formation of foam cells [84]. Platelet exosomes can alleviate coronary artery thrombosis M1-like macrophage polarization via inhibiting miR-34a-5p expression and promoting the decreases in IL-1β, IL-6, and TNF-α [85]. Platelet-rich plasma-derived exosomes inhibit the polarization of M1-like macrophages and promote the polarization of M2-like macrophages. Also, they inhibit the activation of the NLRP3 inflammasome and promote its degradation to suppress the release of IL-1β through the autophagy–lysosome pathway [86]. Polarization towards an M2-like macrophage is suggested to be through the TGF-β signaling pathway promoting the interaction of angiogenesis-, collagen synthesis-, and cell adhesion- and migration-related pathways [87]. Future research is required that focuses on evaluating M2-like macrophage-derived exosomes in inflammatory activity, remodeling, and structural changes in the heart in preclinical models and subsequent clinical trials.

## 11. Therapeutic Potential of Exosomes

Following an MI, various events are triggered, including necrosis, inflammation, and remodeling. Macrophages are involved in all these processes, ultimately leading to cardiac homeostasis. However, excessive cardiac tissue damage, combined with a poorly regulated macrophage response, can trigger undesirable events such as fibrosis, which can lead to heart failure and, consequently, death. Exosomes are emerging as ideal candidates for the treatment of cardiovascular diseases. In recent years, they have been investigated in clinical trials, including exosomes derived from human cells and samples, as well as plant specimens [88]. Exosomes for therapeutic application purposes have focused mainly on those derived from MSCs. They primarily exert therapeutic effects in cardiovascular diseases through paracrine mechanisms mediated by exosomes. These mechanisms involve reducing apoptosis and decreasing autophagic damage in myocardial cells [89]. MSC-derived exosomes in preclinical studies significantly reduced infarct size in rats [90]. Administration of MSCs from the exosome to mice through intramyocardial injection after myocardial I/R reduced infarct size and inflammation in the heart, and also induced the polarization status of M2-like macrophages [91]. Additionally, these exosomes restore bioenergetics, reduce oxidative stress, and activate pro-survival signaling, thereby enhancing cardiac function and geometry after myocardial I/R injury [92]. Preclinical studies in mice show an increase in mir-155 in cardiac tissue after infarction. Mir-155 is highly expressed in tissue-infiltrating macrophages compared to other cell types. Interestingly, macrophages transfer mir-155 to cardiac fibroblasts, decreasing their proliferative capacity and increasing the synthesis of collagen and proinflammatory cytokines such as IL-1β, IL-6, TNF-α, and CCL-2. Thus, macrophage-derived exosomes appear to impair cardiac repair after MI at least partially through the role of mir-155 [93]. M2-like macrophage exosomes enhanced the viability of rat cardiomyocytes while reduced cell apoptosis after I/R. In addition, rats treated with M2-like macrophage exosomes showed a significantly decreased area-at-risk ratio, which indicated that M2-like macrophage exosomes alleviated the I/R injury in cardiac tissues. M2-like macrophage exosomes present a high expression of miR-148a and are little expressed in the I/R. M2-like macrophage exosomes protect injury by means of miR 148 and the downregulation of thioredoxin-interacting protein, and inactivation of the TLR4/NF-κB/NLRP3 inflammasome signaling pathway is involved in this event [94].

Other studies address cellular genetic modifications. Macrophages were genetically modified to secrete exosomes carrying the *SNHG12* gene sequence as lncRNA delivery platforms and cerium–macrophage exosomes were constructed by loading hollow cerium oxide nanoparticles (hCeO_2_). ApoE^−/−^ mice assays demonstrated that cerium–macrophage exosomes targeted the loaded lncRNA *SNHG12* and hCeO_2_ to the atherosclerotic site through membrane proteins inherited from the macrophages. Furthermore, the hCeO_2_ and *SNHG12* loaded into the Ce-Exo exhibited a synergistic effect, improving the inflammatory microenvironment through the decrease in intracellular ROS levels and facilitating DNA repair in endothelial cells, effectively postponing the progression of atherosclerosis [95].

In addition to preclinical studies, clinical studies have also been conducted exploring the therapeutic effects of exosomes in humans (Table 2). However, none of these trials is completed and no results are currently available. The evidence is promising for the use of exosomes from macrophages or other cells as well as genetically modified exosomes in clinical trials. However, further evidence is still required to show considerable benefits in terms of pharmacokinetics, targeting, and safety compared to synthetic nanocarriers [88,96]. Because of the characteristic complexity of the exosomes themselves, size heterogeneity and natural discrepancies run throughout their assembly, and the intrinsic risks of the biogenesis procedure are higher than those of virginally synthetic fabrication approaches [88,97,98].

## 12. Conclusions

Atherosclerosis is a chronic inflammatory disease that, in advanced stages, causes acute myocardial infarction, which can be catastrophic for patients. Macrophages are key cells in the development and progression of atherosclerosis, as well as in infarction, through reparative or injurious responses in cardiac tissue. The polarization of macrophages is crucial in the progression or regression of CVDs. Various key mechanisms can influence this process, such as communication between macrophages and exosomes. Exosomes derived from various cells are excellent messengers because of the stability of their biological contents, such as miRNAs, which play a central role in modulating macrophage polarization, as they can activate or suppress the expression of genes that determine the macrophage phenotype. Exosomes derived from macrophages or other immunoregulatory cells may reduce or control damage in atherosclerosis and after infarction. Exosomes represent a promising new tool for use in CVDs and their complications.

## Figures and Tables

**Table 1:** MiRNAs related to cardiovascular diseases.

Exosome Type	Nucleic Acid	Related CVD	References
Macrophages-EXO	miR-155	Atherosclerosis	[49]
Macrophages-EXO	miR-146a		[50,51]
Macrophages-EXO	miR-21-3p		[52]
mesenchymal stem cells-EXO	miR-let-7		[53]
AdET-1-HUVEC-EXO	miR-33		[54]
Monocytes-EXO	miR-4532		[56]
Macrophages-EXO	miR-146b, miR-378a, and miR-99a		[57]
Endothelial cell-EXO	LncNEAT1		[58]
Endothelial cell-EXO	miR-10a		[59]
mesenchymal stem cells-EXO	miR-182	Ischemia-reperfusion	[60]
mesenchymal stem cells-EXO	miR-181a		[61]
Cardiospheres-EXO	miR-27a-5p, miR-182, and miR-101a		[62]
Plasma-EXO	miR-26b-5p		[63]
stem cell-EXO	miR-21	Ischemia	[64]
Macrophages-EXO	miR-29a	Hypoxia	[65]
Cardiomyocytes-EXO	miR-146a-5p	Myocardial infarction	[66]
Ferroptotic cardiomyocyte-EXO	miR-106b-3p		[67]
Macrophages-EXO	miR-155		[68]
Macrophages-EXO	miR-132-3p		[69]
Macrophages-EXO	miR-155		[59]

**Table 2:** Exosome-based clinical trials.

Exosomes	Condition	Cargo	Phase	Number
MSCs	Familial hypercholesterolemia	Ldlr mRNA	I	NCT05043181
MSCs	Cerebrovascular disorders	miRNA-124	I	NCT03384433
